# What is the Cost of Diagnosis and Management of Drug Resistant Tuberculosis in South Africa?

**DOI:** 10.1371/journal.pone.0054587

**Published:** 2013-01-18

**Authors:** Anil Pooran, Elize Pieterson, Malika Davids, Grant Theron, Keertan Dheda

**Affiliations:** Lung Infection and Immunity Unit, Division of Pulmonology and UCT Lung Institute, Department of Medicine, University of Cape Town, Cape Town, South Africa; Public Health Agency of Barcelona, Spain

## Abstract

**Background:**

Drug-resistant tuberculosis (DR-TB) is undermining TB control in South Africa. However, there are hardly any data about the cost of treating DR-TB in high burden settings despite such information being quintessential for the rational planning and allocation of resources by policy-makers, and to inform future cost-effectiveness analyses.

**Methodology:**

We analysed the comparative 2011 United States dollar ($) cost of diagnosis and treatment of drug sensitive TB (DS-TB), MDR-TB and XDR-TB, based on National South African TB guidelines, from the perspective of the National TB Program using published clinical outcome data.

**Principal Findings:**

Assuming adherence to national DR-TB management guidelines, the per patient cost of XDR-TB was $26,392, four times greater than MDR-TB ($6772), and 103 times greater than drug-sensitive TB ($257). Despite DR-TB comprising only 2.2% of the case burden, it consumed ∼32% of the total estimated 2011 national TB budget of US $218 million. 45% and 25% of the DR-TB costs were attributed to anti-TB drugs and hospitalization, respectively. XDR-TB consumed 28% of the total DR-TB diagnosis and treatment costs. Laboratory testing and anti-TB drugs comprised the majority (71%) of MDR-TB costs while hospitalization and anti-TB drug costs comprised the majority (92%) of XDR-TB costs. A decentralized XDR-TB treatment programme could potentially reduce costs by $6930 (26%) per case and reduce the total amount spent on DR-TB by ∼7%.

**Conclusion/Significance:**

Although DR-TB forms a very small proportion of the total case burden it consumes a disproportionate and substantial amount of South Africa’s total annual TB budget. These data inform rational resource allocation and selection of management strategies for DR-TB in high burden settings.

## Introduction

Tuberculosis (TB) remains a major public health crisis in sub-Saharan Africa despite declining global TB incidence rates [1]. Achieving the United Nations Millennium Development goal to reduce the burden of TB by 50% in 2015 seems unlikely in this region [2]. This is due to several reasons including unsuccessful treatment programmes, the HIV epidemic, increasing economic deprivation and the emergence of drug resistant TB (DR-TB) [3], [4]. Multidrug-resistant TB (MDR-TB), defined as culture-confirmed resistance to rifampicin and isoniazid, comprises ∼3% of new and retreatment TB cases in Africa [1]. Approximately 5 to 10% of all MDR-TB cases are extensively-drug-resistant TB (XDR-TB), defined as MDR-TB plus additional culture-confirmed resistance to a fluoroquinolone and an injectable agent (2^nd^ line aminoglycoside or capreomycin) [3]. The situation, fuelled by high transmission rates and HIV co-infection, is particularly dire in South Africa which has the one of the highest TB incidence rates and the 5^th^ highest DR-TB burden globally [1], [5].

Compared to drug-susceptible TB (DS-TB), MDR-TB and XDR-TB requires longer, more toxic treatment, and is associated with poorer outcomes (less than 20% of XDR-TB cases culture-convert in South Africa [6]–[8] compared to other high burden settings where culture conversion rates were higher [9]). Drug costs for treatment of DR-TB are considerably higher and divert resources away from managing a national TB program (NTP). In 2011, the NTP budget in South Africa was approximately US$218 million and a crude preliminary estimate suggests that almost half was allocated to managing MDR-TB [1], [10]. More accurate per case and total estimates are required by NTPs and policy makers for rational planning and allocation of resources, to determine optimal preventative and management strategies, to prioritise competing health care issues, and to inform future cost-effectiveness analyses. These data are also relevant to the proposed scaling up of TB diagnostic capacity using nucleic acid amplification platforms such as Xpert MTB-RIF (Cepheid, USA), and Genotype MTBDR*plus* and MTBDR*sl* assays (Hain Lifesciences, Germany) as such tests are likely to sharply increase the number of newly diagnosed cases of DR-TB [11]. However, there are limited data globally about the cost of treating multi-drug resistant TB [12]–[17] and none about management-related costs in South Africa. Furthermore, there are no studies that have directly assessed the cost of XDR-TB in South Africa or elsewhere.

To address these gaps in our knowledge we performed a comprehensive cost analysis of MDR-TB and XDR-TB in the Western Cape province of South Africa, based on the current national DR-TB guidelines. As TB treatment costs in different provinces are similar, our analysis reflects costs of DR-TB treatment in South Africa in general. Additionally, we evaluated the costs of a hypothetical decentralized treatment programme for XDR-TB that could potentially reduce the financial burden on South Africa’s healthcare system.

## Methods

We performed a cost analysis to determine the economic impact of DR-TB on the National TB Programme in South Africa. The analysis was performed from the perspective of the South African National TB Program which incurs all TB related management costs, including ADR management, surgery, drugs, hospitalization and diagnostic/monitoring tests. Strict adherence to National South African DR-TB management guidelines was assumed in the analysis. All direct and indirect medical and non-medical costs were included for the year 2011. The time horizon for the analysis was 6 months for DS-TB and 24 months for DR-TB, which is the length of a full course of anti-DS-TB and anti-DR-TB treatment, respectively. Future costs were adjusted for inflation using the South African Consumer Price Index where appropriate [18]. All costs were expressed in 2011 $US at an exchange rate of $1USD = ZAR7.05 [19]. Estimates of DS-TB, MDR-TB and XDR-TB disease outcomes were taken from published cohort studies specifically conducted in South Africa [1], [6]–[8], [20]–[30]. Component costs are shown in table 1.

**Table 1:** Costs of components associated with diagnosis and treatment of drug sensitive, multi-drug resistant and extensively drug resistant tuberculosis.

Cost Component	Cost ($US)	Source
**Inpatient stay (per day)**		BCH expenditure reports, Department of Health, BCH statistical reports, staff interviews
Capital costs (Buildings and Equipment)	$4.70	
Nursing & Medical staff	$18.42	
Support staff (OT, PT, Dietician, Psychologist, Data Capturer, etc)	$4.77	
Administrative staff	$2.93	
Staff overhead (excluding administrative staff)	$7.20	
Ancillary (Kitchen & Laundry)	$9.23	
Recurrent medical consumables	$3.91	
Non personnel recurrent overheads (utilities and other general supplies)	$4.93	
**Total**	**$56.07**	
**Outpatient visit (per visit)**		BCH expenditure reports, Department of Health, BCH statistical reports, staff interviews
Capital costs (Buildings and Equipment)	$1.59	
Nursing & Medical staff	$6.13	
Support staff (OT, PT, Dietician, Psychologist, Data Capturer, etc.)	$7.15	
Administrative staff	$0.97	
Staff overhead (excluding administrative staff)	$2.39	
Recurrent medical consumables	$1.30	
Non personnel recurrent overheads (utilities and other general supplies)	$1.64	
**Total**	**$21.16**	
**Clinic visit (per visit)**		Langa and Chapel St Clinic expenditure reports, Cape Town City Health, clinic staff interviews
Capital costs (Buildings and Equipment)	$0.61	
Staff (Nursing, Medical and General administrative staff)	$4.44	
Recurrent medical consumables	$0.90	
non personnel recurrent overheads (utilities and other general supplies)	$0.69	
**Total**	**$6.64**	
**10 minute DOTS worker visit (per visit)**	$1.10	Provincial government salary scales
**Drugs**		BCH pharmacy, Cape Town City Health
DS-TB		
2 mth Intensive (RHZE)	$16.77	
4 mth Continuation (RH)	$20.96	
DS-TB Retreatment		
3 mth Intensive (RHZES)	$135.45	
5 mth Continuation (RHE)	$53.03	
MDR-TB		
6 mth Intensive (Km-Z-Mxf-Eto-Trd)	$1,438.03	
18 mth Continuation (Z-Mxf-Eto-Trd)	$3,671.48	
XDR-TB Primary		
6 mth Intensive (Cm-Z-Mxf-Eto-Trd-PAS-Cfz)	$5,272.91	
18 mth Continuation (Cm-Z-Mxf-Eto-Trd-PAS-Cfz)	$15,015.62	
XDR-TB Acquired		
6 mth Intensive (Cm-Z-Mxf-Clm-Aug-hdH-PAS-Cfz)	$4,884.80	
18 mth Continuation (Z-Mxf-Clm-Aug-hdH-PAS-Cfz)	$13,406.14	
**Tests**		National Health Laboratory Services, Groote Schuur Hospital, WHO
Auramine Fluorescent Smear Microscopy	$3.71	
MGIT 960 liquid Culture	$14.02	
Xpert MTB-RIF	$21.39	
1st line DST (Line Probe Assay)	$26.74	
2nd line DST for 4 drugs (MGIT 960 liquid culture)	$37.83	
Chest X-ray	$31.91	
Audiogram	$25.60	
Liver function (Potassium, Urea, Creatinine)	$15.05	
Kidney function (ALT, AST, Bilirubin)	$10.86	
Thyroid function	$20.89	
HIV Rapid screening test	$5.26	
Other HIV associated tests (CD4 count, viral load)	$51.75	
**Specimen transport**		Department of Transport, BCH expenditure reports, staff interviews
Capital	$0.28	
Recurrent cost	$1.03	
Personnel	$1.40	
**Total**	**$2.72**	
**Surgery**		Groote Schuur Hospital
Pneumonectomy	$5,549.36	
**Death** i.e. Removal of body from premises	$70.92	BCH financial department

DS-TB - Drug sensitive tuberculosis, MDR-TB – Multi-drug resistant tuberculosis, XDR-TB – Extensively Drug-resistant tuberculosis, ADR - Adverse Drug reaction, OT - Occupational Therapist, PT - Physiotherapist, BCH – Brooklyn Chest Hospital, DOTS – Directly Observed Treatment Short Course, MGIT – Mycobacterial Growth In-tube, DST – Drug susceptibility test, AST - aspartate aminotransferase, ALT - alanine aminotransferase, R - Rifampicin, H - Isoniazid, Z - Pyrazinamide, E - Ethambutol, S - Streptomycin, Km – Kanamycin, Mxf - Moxifloxacin, Eto - Ethionamide, Trd – Terizidone, Cm - Capreomycin, PAS – para-amionsalicylic acid, Cfz – Clofazimine, Clm-Clarithromycin, Aug - Augmentin, hdH - high dose Isoniazid.

### Unit Costs

#### Inpatient, outpatient and clinic costs

The Western Cape Province has the third highest incidence of DR-TB in South Africa and accounts for 15% of DR-TB cases [5]. Brooklyn Chest Hospital (BCH) is a designated DR-TB hospital which provides inpatient and outpatient services for the majority of MDR-TB and XDR-TB patients in the Western Cape. Hospital-associated costs were provided by BCH and are assumed to be representative of this type of facility in South Africa. Inpatient and outpatient costs were calculated using a WHO standardised tool for economic analyses [31]. Personnel costs, including nursing, medical, patient support and other general staff, were obtained from provincial government salary scales and included basic salaries, medical aid contributions and housing allowances. Capital costs included all buildings, equipment, vehicles and furniture. Building and vehicle costs were calculated based on the current replacement costs at BCH, according to standard protocols [31]. Capital costs were annualized at a discount rate of 3%. The useful lifetime was assumed to be 50 years for buildings and 10 years for vehicles and equipment. Outpatient services were assumed to use a proportion of hospital overhead costs determined by standard methods [31]. Medical consumables and other recurrent overhead costs, including utilities and maintenance costs were obtained from 2011 BCH expenditure reports. Ancillary costs, including kitchen and laundry services, were attributable to inpatient costs only and were also obtained from BCH expenditure reports. Outpatient services were assumed to use a proportion of hospital overhead costs [31]. Total inpatient and outpatient costs were divided by the total number of BCH inpatient days and outpatient visits to calculate the cost per inpatient day and outpatient visit, respectively. In South Africa, the majority of DS-TB cases are diagnosed and treated at primary care clinics under the National Directly Observed Treatment Short-Course (DOTS) Programme. Primary care clinic visit costs were calculated, in a similar manner to inpatient and outpatient costs, using data from two TB clinics located in the Cape Town Metro region, Langa Clinic and Chapel Street Clinic. An inpatient day at BCH costs $56.07 whereas an outpatient visit costs $21.16. The cost of a primary care clinic visit (average of two clinics) was $6.64. The cost of a DOTS clinic visit was $1.10 (DS-TB). M/XDR-TB and DS-TB retreatment patients incur a higher DOTS clinic visit cost as they require a nurse for administering injectable drugs ($2.01 per visit).

#### Drug costs

Drug costs were based on current government tender prices and include those prescribed in standard treatment regimens for DS-TB, MDR-TB and XDR-TB. DR-TB regimens are based on country specific drug resistance profiles and use of second line drugs [5]. DS-TB drugs include four first line agents isoniazid, rifampicin, ethambutol and pyrazinamide as well as streptomycin for retreatment cases. MDR-TB and XDR-TB require much longer treatment duration using second and third line anti-tuberculosis agents. MDR-TB patients use kanamycin in the intensive treatment phase and ethionamide, pyrazinimide, moxifloxacin and terizidone in both the intensive and continuation phase. Primary XDR –TB patients are treated with capreomycin in the intensive phase and ethionamide, pyrazinimide, moxifloxacin, clofazimine, terizidone and para-aminosalycilic acid (PAS) in both intensive and continuation phases. Acquired XDR-TB patients substitute ethionamide and terizidone for augmentin, clarithromycin and high dose isoniazid in their treatment regimen. HIV ARV drug costs have been excluded from the analysis as the incremental costs would be zero for MDR-TB, XDR-TB and DS-TB if HIV ARV therapy was assessed for an equivalent period. However, the inclusion of HIV ARV costs was assessed in the sensitivity analysis. Total treatment regimen costs were calculated based on these standardised regimens for a 50–70 kg patient given treatment 6 days a week.

#### Diagnostic and monitoring test costs

The costs of all laboratory based tests, for bacteriological assessment, drug susceptibility testing (DST), HIV and adverse drug reaction (ADR) monitoring were provided by the National Health Laboratory Service (NHLS). The NHLS is a reference lab which provides services for the public healthcare system so these costs represent the actual costs incurred by the NTP. HIV monitoring test costs were included in the analysis according to current DR-TB management guidelines [5]. Test costs obtained directly from the NHLS have been used in previous health economic analyses [11], [32]. The cost of a chest X-ray (CXR) was obtained from Groote Schuur Hospital (a CXR referral centre for primary care clinics in Cape Town). The cost of an audiogram was calculated from BCH data using an ingredient’s approach. The cost of an Xpert MTB-RIF was calculated from WHO estimates [33] and South African specific data using an ingredient’s approach. Specimen transport costs were calculated separately using data from BCH according to standard protocols [31] and incorporated in the total test costs. Total test costs were determined by multiplying unit costs provided by the NHLS by the frequency of tests performed for the period of treatment as recommended in the national TB and Drug Resistant TB policy guidelines [5], [34] (Table S1).

#### Adverse drug reaction (ADR) costs

An ADR, due to either first-line or second-line anti-TB drugs, was assumed to be 3 weeks in duration and the total cost included that of ancillary drugs, extra weekly monitoring tests and hospital outpatient or clinic visits during this timeframe, in accordance with national guidelines [5], [34], [35]. The incidence of ADRs associated with DS-TB, MDR-TB and XDR-TB treatment was taken from the literature [6], [25], [26]. Additionally, we assumed that some patients who develop severe ADRs [30% for MDR-TB and XDR-TB [6], [29]; 10% for DS-TB [25]] will require hospitalization for 2 weeks [36]. Given that first line drugs are associated with less severe ADRs [37] and DS-TB patients are generally not as sick as DR-TB patients, we assume that DS-TB associated ADRs only require 1 week hospitalization [38]. We did not account for the reduction in costs associated with lowering the dosage or withdrawing TB drugs and we assumed that substitution of drugs will occur at no additional cost [39]. Additionally, we assumed the same ADRs occur with the same frequency in HIV infected and HIV uninfected individuals.

#### Surgery costs

The cost of a pneumonectomy procedure was used to represent the cost of surgical resection in DR-TB cases. This cost was provided by the Groote Schuur Surgical Department and included the costs of facilities, medication, personnel and tests associated with surgery.

#### Death-related costs

Death-related costs were only incurred by inpatients that died in hospital and included the cost for removal of the body from the hospital premises. We assumed all other death related costs to be borne by the patient.

### Diagnostic and Treatment Algorithms

Protocols for the diagnosis and treatment of DS-TB, MDR-TB and XDR-TB were followed according to South African National Department of Health Guidelines [5], [34]. Treatment associated outcomes have been defined in line with these guidelines. Only new and retreatment cases of pulmonary DS-TB and new cases of MDR-TB and XDR-TB were considered in the analysis. All outcome probabilities are shown in Table 2.

**Table 2:** Per case probability estimates of different diagnosis- and treatment-related outcomes for drug sensitive, multi-drug resistant and extensively-drug-resistant tuberculosis.

Outcome	DS-TB		MDR-TB		XDR-TB	
	estimate	source	estimate	source	estimate	source
**Diagnostic outcomes and assumptions**						
HIV prevalence	0.50	[1]	0.60	[43]	0.60	[6]–[8]
Proportion of smear positives	0.6	[44]	Not included*			
Proportion of smear negatives	0.21	[44]	Not included*			
Proportion of retreatment cases	0.19	[44]	Not included*			
**Treatment outcomes and assumptions**						
Proportion treated as hospital inpatients	0	Assumed†	0.10	Assumed‡	1.00 0.5	[5] Assumed§
Proportion treated as hospital outpatients/PCC	1.00	Assumed†	0.90	Assumed‡	0 0.5	[5] Assumed§
Duration of treatment (assuming treatment completion)	6 months	[34]	2 years	[5]	2 years	[5]
Proportion who culture convert and complete treatment	Not included¶		0.50	[22], [23], [29]	0.20	[6], [8]
Average time from diagnosis to culture conversion	Not included¶		4 months	[23], [29]	6 months	[6], [7]
Death during Treatment	0.05	[44]	0.20	[21], [22], [29]	0.40	[6]–[8]
Average time from diagnosis to death	3 months	[13]	5 months	[20], [21]	7 months	[6]
Treatment default	0.1	[44]	0.2	[20]	Assumed same as MDR-TB	
Time to treatment default	3 months	[13]	5 months	[13]	Assumed same as MDR-TB	
Treatment failure	Not included¶		0.10	[22], [29]	0.40	[6]
Average time from diagnosis to treatment failure &discharge from hospital	Not included¶		12 months	Assumed #	12 months	[6]
Proportion developing ADRs	0.05	[25]	0.30	[26]	0.60	[6]
Proportion of patients developing ADRs that arehospitalized	0.10	Assumed, [25]	0.30	[29]	0.60	[6]
Proportion undergoing surgery	0.00	Assumed	0.02	Assumed #	0.02	[6], [24]

* These proportions were not included in the model as all MDR-TB and XDR-TB cases follow the same diagnosis and treatment protocols regardless of smear status in accordance with national guidelines. Additionally, we only modelled new cases of MDR-TB and XDR-TB and did not include retreatment cases.

† Assumed that all DS-TB patients are treated at a primary care clinic and none are hospitalized.

‡ This estimate was provided by Brooklyn Chest Hospital.

§ A figure of 50% is assumed for the decentralized XDR-TB model based on a proportion of patients from an XDR-TB cohort who weigh >50 kg,

¶ Not incorporated into our model as we assume all DS-TB patients complete a full course of treatment, whether they are cured or they fail treatment.

# Assumed to be the same as XDR-TB.

DS-TB - Drug sensitive tuberculosis, MDR-TB – Multi-drug resistant tuberculosis, XDR-TB – Extensively Drug-resistant tuberculosis, PCC - primary care clinic, ADR - Adverse Drug reaction.

#### DS-TB

DS-TB patients are initially assessed and diagnosed at a primary health care clinic and are followed up at subsequent visits. Bacteriological monitoring tests are performed at diagnosis and periodically during treatment, according to National TB guidelines (Table S1). Xpert MTB-RIF is also performed on all TB and DR-TB suspects at initial diagnosis only, in line with current recommendations [5]. Treatment is initiated under the DOTS program for the first two weeks at the clinic followed by community-based DOTS treatment. The standard DS-TB treatment regimen includes a 2-month intensive phase using 4 drugs (isoniazid/rifampicin/ethambutol/pyrazinamide) followed by a 4-month continuation phase (isoniazid/rifampicin). Retreatment cases require 3 months of intensive phase treatment with streptomycin included in the regimen, followed by a 5-month continuation phase (isoniazid/rifampicin/ethambutol). Patients who do not sputum convert (i.e. become smear negative) after the initial intensive phase of treatment receive an extra month of intensive phase treatment. We assume that DS-TB patients who die or default from treatment only incur half the cost of a treatment regimen [13]. Furthermore, as the vast majority of DS-TB cases are treated in the community, we assume that no DS-TB patients are hospitalized.

#### MDR-TB

Decentralized MDR-TB treatment in South Africa was initiated in 2002, under the WHO DOTS-Plus program. The national DR-TB guidelines recommend that the majority of cases be treated as outpatients and only severely ill MDR-TB patients should be admitted for hospitalization (10% of cases). Diagnostic and monitoring tests are performed (Table S1) as recommended in the DR-TB management guidelines. We assume HIV infected patients undergo six-monthly assessments of CD4 counts and viral loads for the duration of TB treatment. A standardized MDR-TB regimen consists of a 6-month intensive phase of 5 drugs (Ethionamide/Pyrazinimide/Kanamycin/Moxifloxacin/Terizidone) followed by an 18-month continuation phase with 4 drugs (Ethionamide/Pyrazinimide/Moxifloxacin/Terizidone). In addition to monthly hospital outpatient facility visits for scheduled medical checkups, MDR-TB outpatients attend local TB clinics daily to receive their drugs and monitoring for development of ADRs. Inpatients remain hospitalized until they culture convert (i.e. have two successive months of negative sputum cultures), then continue treatment as outpatients, including daily attendance at TB clinics for drugs and ADR monitoring during the continuation phase of treatment. If patients do not culture convert after 12 months of treatment, they are considered treatment failures. We assume these ‘non converters’ will be referred for XDR-TB treatment and no longer incur MDR-TB associated costs.

#### XDR-TB

In South Africa, national DR-TB management guidelines recommend hospitalization of all confirmed XDR-TB patients. A standardized regimen for primary XDR-TB (25% of XDR-TB cases [6], [7]) consists of a 6–month intensive phase of 7 drugs (Ethionamide/Pyrazinimide/Capreomycin/Moxifloxacin/Clofazimine/Terizidone/Para-aminosalycilic acid (PAS)) followed by an 18-month continuation phase of 6 drugs (Ethionamide/Pyrazinimide/Moxifloxacin/Clofazimine/Terizidone/PAS). An acquired XDR-TB (75% of XDR-TB cases) treatment regimen is the same except that ethionamide and terizidone are substituted for augmentin, clarithromycin and high dose isoniazid. Clinic visits, bacteriological, radiological and HIV monitoring tests are performed with the same frequency as during MDR-TB management. However, due to the increased toxicity of XDR-TB drugs, such as capreomycin and PAS, we assume certain ADR monitoring tests (kidney function tests, thyroid function test) are performed more frequently (Table S1). In South Africa, provincial review boards decide on whether or not to stop treatment in patients who fail to culture convert after 12 months of XDR-TB treatment. Based on clinical guidance, we assume that these ‘treatment failures’ are discharged from hospital after 12 months but receive an extra 3 months of continuation phase treatment before a review board decides to stop treatment completely.

#### Alternative XDR-TB decentralization programme

Due to resource constraints on centralized treatment facilities, we propose a decentralized programme where XDR-TB treatment is initiated in an outpatient facility and follow similar protocols to the decentralized MDR-TB strategy. We estimate that approximately 50% of XDR-TB cases will be suitable for ambulatory care (based on clinical experience and estimated by the proportion of XDR-TB patients who weigh <50 kg at diagnosis, which has shown to be associated with worse treatment outcomes [6]). However, in the absence of regulatory guidelines regarding the monitoring frequency of XDR-TB outpatients and due to the poorer XDR-TB outcomes, we assume these patients will be monitored more closely than in MDR-TB. This includes hospital outpatient visits twice a month during the intensive phase and monthly for the continuation phase. We assume that monitoring tests will be conducted with the same frequency as hospitalized patients and XDR-TB drugs will continue to be dispensed at local TB clinics for the entire course of treatment.

### Sensitivity Analysis

A univariate sensitivity analysis was performed to measure uncertainties in the component costs and outcome data used in the analysis. Parameters that were varied include cost and length of hospitalization, length of treatment, discount rate for capital items, ADR prevalence, the incidence of surgery and the proportion of MDR-TB and XDR-TB patients hospitalized. Additionally, the inclusion of HIV ARV drug costs and HIV prevalence were varied in the sensitivity analysis. HIV ARVs costs were determined for the period of TB treatment and included 5 standard first-line drugs (Efavirenz/Stavudine/Lamuvidine/Co-Trimoxizole/Pyridoxine) given seven days a week.

Despite decentralized MDR-TB treatment being the current policy in South Africa, many provinces still hospitalize the majority of MDR-TB patients until culture conversion. We therefore perform a sensitivity analysis to determine the per patient and total national costs of MDR-TB assuming a higher rate of hospitalization among MDR-TB patients (70% in South Africa according to the WHO [5]).

## Results

**Figure 1:**
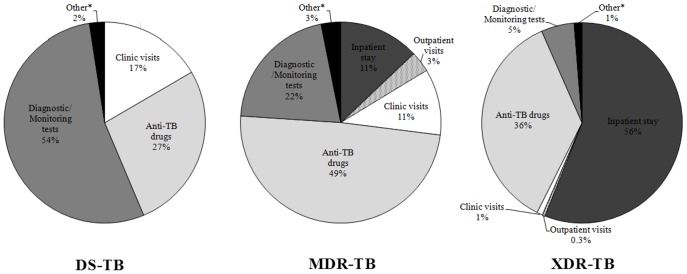
The cost breakdown of the total cost per patient for drug sensitive (DS-TB), multi-drug resistant (MDR-TB) and extensively drug-resistant (XDR-TB) tuberculosis. *Other indicates surgery, ADRs and death related costs.

TB treatment costs and cost breakdown are shown in table 3. The cost per case of smear-positive DS-TB was $191.66 whereas the cost of a smear-negative and retreatment case was more expensive, at $252.54 and $455.50 respectively, mainly due to the increased number of diagnostic tests and the longer and more expensive retreatment regimen. When the proportion of cases in each category (smear positive, smear negative, retreatment), based on 2010 TB case findings [1], was multiplied by the total costs of each category, the overall cost of DS-TB was $256.61. The cost of MDR-TB was much more expensive than DS-TB, at $5,930.02 for an MDR-TB outpatient and $14,348.94 for an MDR-TB inpatient. We assumed that approximately 90% of MDR-TB cases are treated as outpatients (according to national DR-TB guidelines) while the remainder are hospitalized due to severe illness. Based on these proportions, the overall cost per case of MDR-TB was $6,771.92. This cost was twenty-six times greater than DS-TB with 49% of these costs being attributable to anti-MDR-TB drugs (Figure 1). Conversely, all confirmed XDR-TB cases require hospitalization. Management of an XDR-TB case costs $26,392.01, four times greater than the cost of an MDR-TB case and 103 times greater than that of a DS-TB case. While XDR-TB drugs do make up a significant proportion of these costs (36%), hospitalization contributes to 56% of the total XDR-TB costs (Figure 1).

**Table 3:** Total costs and breakdown per patient for drug sensitive tuberculosis, multi-drug resistant tuberculosis and extensively drug-resistant tuberculosis. Costs are expressed in $US.

Cost Components	Drug Sensitive TB			MDR-TB		XDR-TB	
	Smear Positive	Smear negative	Re-treatment	Outpatient Treatment	Inpatient treatment	Outpatient Treatment	Inpatient treatment
Hospital inpatient stay	$0.00	$0.00	$0.00	$0.00	$8,746.99	$0.00	$14,802.60
Hospital outpatient visit	$0.00	$0.00	$0.00	$240.22	$130.16	$524.46	$105.40
PCC visit	$32.85	$37.49	$76.98	$749.54	$498.08	$724.39	$240.82
Anti-TB drugs	$40.24	$37.73	$188.48	$3,321.70	$3,321.70	$9,501.57	$9,501.57
Diagnostic/Monitoring Tests	$112.30	$171.05	$183.77	$1,408.21	$1,413.28	$1,477.66	$1,409.97
							
ADRs	$6.27	$6.27	$6.27	$99.37	$99.37	$192.29	$192.29
Surgery	$0.00	$0.00	$0.00	$110.99	$110.99	$110.99	$110.99
Death	$0.00	$0.00	$0.00	$0.00	$28.37	$0.00	$28.37
**TOTAL**	**$191.66**	**$252.54**	**$455.50**	**$5,930.02**	**$14,348.94**	**$12,531.36**	**$26,392.01**
**Prevalence in each group**	**0.60**	**0.20**	**0.20**	**0.90**	**0.10**	**0.00** * **0.50**	**1.00** * **0.50**
**Overall cost (group prevalence x cost per patient)** †	**$256.61**			**$6771.92**		**Current strategy** * **$26,392.01** **Decentralized strategy $19,461.68**	

* In the current model 100% of XDR-TB patients are hospitalized whereas in the proposed decentralized model 50% are treated as outpatients and the remaining 50% are hospitalized.

† For example, the total costs of illness arising from DS TB per patient were calculated as (0.6*191.66)+(0.2*252.54)+(0.2*455.50) = $256.61.

DS-TB - Drug sensitive tuberculosis, MDR-TB – Multi-drug resistant tuberculosis, XDR-TB – Extensively Drug-resistant tuberculosis, PCC- primary care clinic, ADR- Adverse Drug reaction.

**Figure 2:**
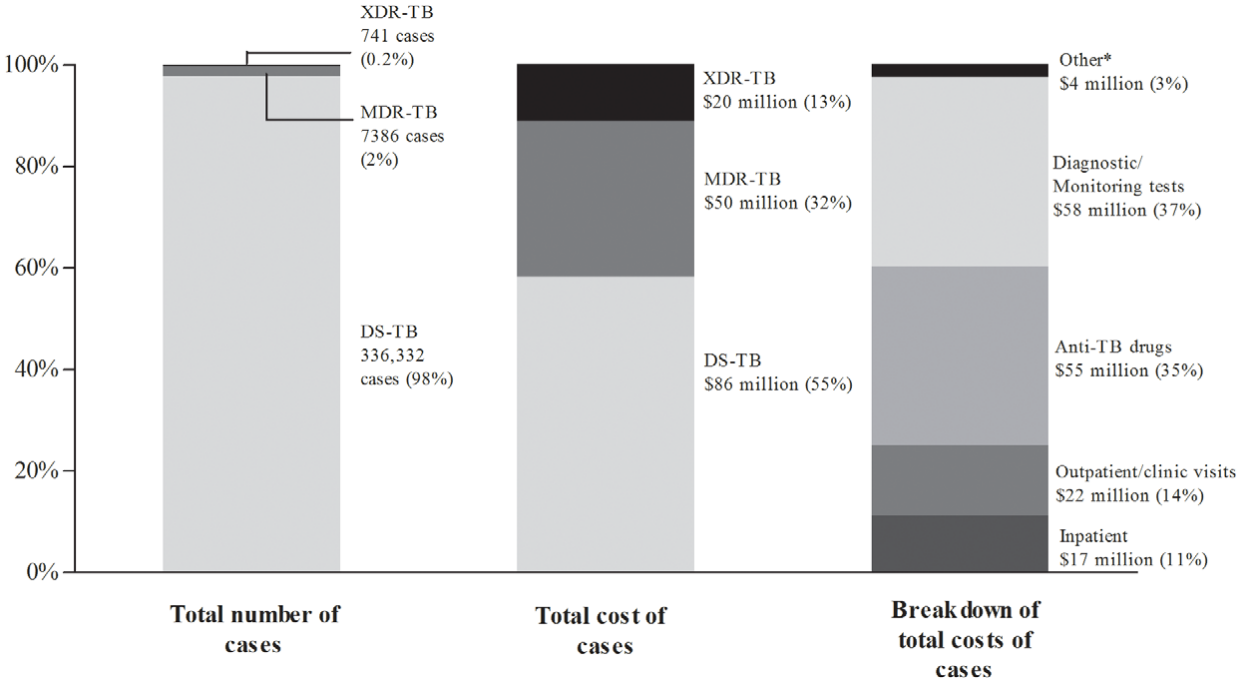
The total number, national costs and cost breakdown of notified cases of drug sensitive (DS-TB), multi-drug resistant (MDR-TB) and extensively drug-resistant tuberculosis (XDR-TB) reported in 2010. Costs are expressed in $US and refer to the cost of diagnosis and treatment of confirmed cases. *Other indicates surgery, ADRs and death related costs.

**Figure 3:**
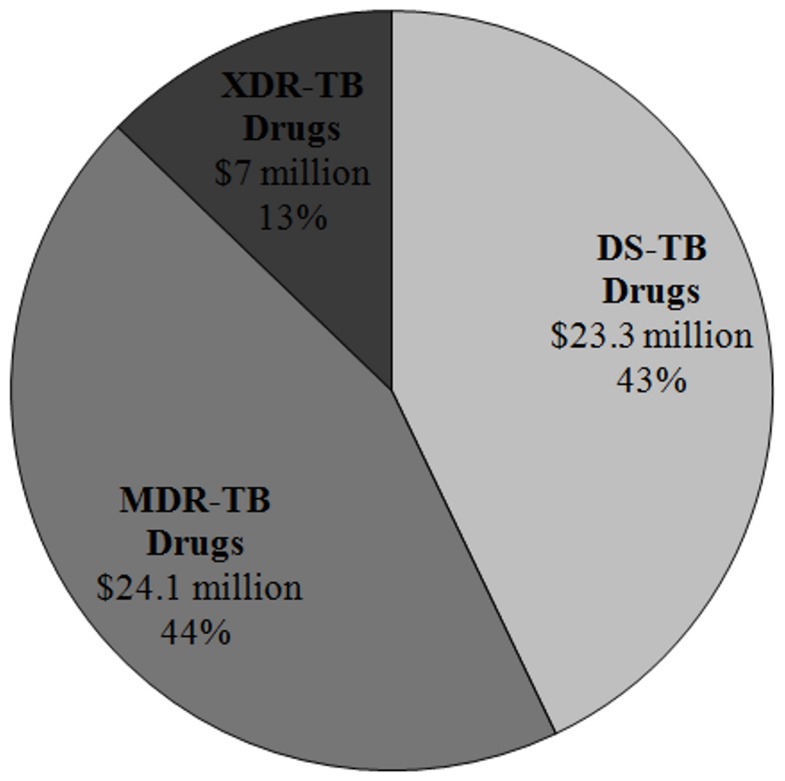
The total drugs costs of notified cases of drug sensitive (DS-TB), multi-drug resistant (MDR-TB) and extensively drug-resistant tuberculosis (XDR-TB) reported in 2010. Costs are expressed in $US.

These costs were applied to a national level, where the total number of notified pulmonary TB cases in 2010 in South Africa [1], [5] was used to obtain a generalized total cost spent on diagnosis and treatment of confirmed TB (Figure 2). In terms of total cases, only a small proportion were MDR-TB and XDR-TB (2% and 0.2%, respectively) but these cases contributed to a significant proportion of the total TB diagnosis and treatment costs (32% and 13%, respectively). Anti-TB drugs is a major contributor to the total cost of TB diagnosis and treatment ($55 million) and DR-TB drugs made up 58% of these costs (Figure 3).

We also proposed an alternative XDR-TB management programme where healthier XDR-TB patients are managed through hospital outpatient facilities and primary care clinics with vigorous follow-up procedures. In this model, hospital costs were eliminated which reduced the cost of diagnosing and treating an XDR-TB case to $12,531.36, a cost saving of 53%. Furthermore, we assume 50% of XDR-TB patients are suitable for ambulatory care. If these patients are treated in the community while the remainder are still treated as inpatients, then XDR-TB would cost $19,461.68 per case and reduce the cost by $6930 compared to current XDR-TB management practice.

We conducted a sensitivity analysis where model parameters (costs and outcomes) were varied to assess any uncertainty in these variables (Table 4). Inpatient day costs had the most significant influence on XDR-TB costs followed by the proportion of patients treated as inpatients, which is not surprising as hospitalization comprises a substantial proportion of per patient XDR-TB costs. Similarly, the MDR-TB analysis was most sensitive to changes in the proportion hospitalised and duration of treatment. Variation in the proportion of non converters (treatment failures) also caused noticeable changes in overall DR-TB costs, mainly due to the increased length of hospitalization required in these individuals. Overall costs of DS-TB, MDR-TB and XDR-TB changed minimally when other parameters, such as inclusion of HIV ARV drug costs, were varied.

**Table 4:** Sensitivity analysis. Costs represent the cost per case and are expressed in $US.

Variables		DS-TB	MDR-TB	XDR-TB current practice	XDR decentralized strategy
**Baseline**	****	**$256.61**	**$6,771.92**	**$26,392.01**	**$19,461.68**
**Discount rate**					
	10%	**$261.33**	**$6,973.50**	**$28,514.62**	**$20,589.74**
	1%	**$255.55**	**$6,728.00**	**$25,934.11**	**$19,218.09**
**Treatment duration**					
	30 months	**$256.61**	**$7,787.05**	**$27,111.95**	**$20,181.62**
	18 months	**$256.61**	**$5,813.29**	**$25,706.93**	**$18,776.61**
**Cost of Hospitalization/Outpatient visit**					
	doubled	**$260.53**	**$7,965.52**	**$41,479.40**	**$27,357.31**
	halved	**$254.65**	**$6,175.11**	**$18,848.31**	**$15,513.87**
**HIV prevalence**					
	90%	**$279.48**	**$6,825.74**	**$26,430.66**	**$19,500.34**
	30%	**$245.17**	**$6,718.09**	**$26,353.35**	**$19,423.03**
**HIV costs**					
Inclusion of ARVs	(period of TB Treatment)	**$329.84**	**$7,012.12**	**$26,632.21**	**$19,701.88**
**ADR frequency**					
MDR					
	50%	**$256.61**	**$6,838.16**	**$26,392.01**	**$19,461.68**
	10%	**$256.61**	**$6,705.67**	**$26,392.01**	**$19,461.68**
XDR					
	80%	**$256.61**	**$6,771.92**	**$26,456.10**	**$19,525.78**
	30%	**$256.61**	**$6,771.92**	**$26,295.86**	**$19,365.54**
**Surgery**					
	10%	**$256.61**	**$7,215.86**	**$26,835.95**	**$19,905.63**
	1%	**$256.61**	**$6,716.42**	**$26,336.51**	**$19,406.19**
**Time to culture conversion (Length of hospitalization)**					
MDR-TB					
	8 months	**$256.61**	**$7,095.49**	**$26,392.01**	**$19,461.68**
	2 months	**$256.61**	**$6,610.00**	**$26,392.01**	**$19,461.68**
XDR-TB					
	12 months	**$256.61**	**$6,771.92**	**$28,327.12**	**$20,429.24**
	2 months	**$256.61**	**$6,771.92**	**$25,118.86**	**$18,825.11**
**% non converters**					
MDR-TB					
	50%	**$256.61**	**$5,828.11**	**$26,392.01**	**$19,461.68**
	30%	**$256.61**	**$6,300.01**	**$26,392.01**	**$19,461.68**
XDR-TB					
	55%	**$256.61**	**$6,771.92**	**$27,362.82**	**$19,744.69**
	10%	**$256.61**	**$6,771.92**	**$24,450.37**	**$18,895.66**
**% hospitalized**					
MDR-TB					
	50%	**$256.61**	**$10,139.48**	**$26,392.01**	**$19,461.68**
	80%	**$256.61**	**$12,665.16**	**$26,392.01**	**$19,461.68**
XDR-TB					
	90%	**$256.61**	**$6,771.92**	**$26,392.01**	**$25,005.94**
	10%	**$256.61**	**$6,771.92**	**$26,392.01**	**$13,917.42**

DS-TB - Drug sensitive tuberculosis, MDR-TB – Multi-drug resistant tuberculosis, XDR-TB – Extensively Drug-resistant tuberculosis, ADR – Adverse Drug reaction, ARVs – Anti-Retroviral drugs.

Under the assumption of 70% hospitalization among MDR-TB patients, the per patient cost of MDR-TB was $11,823, a difference of $5,051 compared to MDR-TB costs in our primary analysis ($6772 per case). Not surprisingly, inpatient costs comprise the majority of these per patient costs (52%). Furthermore, in this scenario, MDR-TB costs constitute 49% of the total national TB diagnosis and treatment costs (compared to 32% in the primary analysis). Consequently, the national DR-TB diagnosis and treatment costs consume ∼50% of the 2011 NTP budget. Hospitalisation costs comprise the majority of the DR-TB national costs (58%) under this assumption.

## Discussion

We performed a cost analysis to determine the specific costs associated with the diagnosis and treatment of MDR-TB and XDR-TB in South Africa, including hospital inpatient and outpatient, and primary care clinic costs, diagnostic and treatment monitoring costs, drug costs, surgical costs, and costs associated with HIV and ADR management. This is, to our knowledge, the first study to accurately quantify the cost of treating XDR-TB and the first to assess the costs of DR-TB management in South Africa according to the current South African DR-TB guidelines. It will facilitate planning and resource allocation, and will inform future cost-effectiveness studies. Our main findings were (i) despite DR-TB comprising only 2.2% of the total case burden it consumed 32% of the estimated 2011, ∼US$218 million, total national TB budget. (ii) XDR-TB consumed 28% of total DR-TB management costs and 9% of the total national TB budget; (iii) overall, 45% and 25% of DR-TB costs were attributed to anti-TB drugs and hospitalization, respectively (49% and 13% of MDR-TB costs; 36% and 56% of XDR costs, respectively); (iv) a decentralised XDR-TB treatment model could reduce overall DR-TB costs by ∼7% and the XDR-TB-specific costs by ∼26%.

Despite DR-TB cases comprising a trivial proportion of the total case burden they consumed a disproportionately large amount of total TB costs. This was due to the high cost of managing DR-TB. Indeed, despite the small number of total cases of XDR-TB (0.2% of total notified cases in 2010), the cost was disproportionately expensive ($26,392.01 per case and representing 9% of the estimated total national TB costs). High drug prices [40] and the need for extensive supervised patient care contribute to the high cost of diagnosing and treating DR-TB. These costs (as a % of total costs) are likely to increase substantially as the South African Department of Health has recently recommended implementation of the new Xpert MTB/RIF assay as the primary TB diagnostic test in all persons with suspected TB [41]. This test, which also detects rifampicin resistance, will likely increase the detection rate and, consequently, the total costs of DR-TB [11]. Thus, effective and sustainable financial and management strategies will be required to deal with this expected rise in notified MDR-TB (and XDR-TB) cases.

What are the major components driving the high DR-TB costs? XDR-TB despite forming only 0.2% of the total case load consumed ∼26% of DR-TB diagnosis and treatment costs (and ∼9% of total diagnosis and treatment costs). The major drivers here were hospitalisation (56% of total XDR-TB costs) and drug costs (36% of total XDR-TB costs). Drugs such as clarithromycin and augmentin contributed to these costs (4% of total XDR-TB drug costs) despite being of uncertain value in the effective treatment of cases. Such costs could be diverted to other aspects of the programme. By contrast, for MDR-TB laboratory testing and anti-TB drugs comprised the majority (71%) of MDR-TB costs. The MDR-TB per case costs in our study ($6,772) was different compared to Russia (US$14,600) and Peru (US$2400) [17] mainly reflecting differences in hospitalization and drug prices, but also inclusion or exclusion of specific cost components.

A substantial proportion of DR-TB costs were attributed to XDR-TB. Thus, targeting this component of DR-TB could potentially reduce costs substantially. If, in contrast to the existing model where all cases of XDR-TB are admitted to hospital, a decentralized XDR-TB treatment strategy was adopted (where patients receive their drugs and undergo routine clinical assessments at their local TB clinics), costs savings of $6930 per case of XDR-TB could potentially be made depending on the rate of hospitalisation of these patients. These savings would primarily be made through reduced hospitalization costs, which contribute to over half of all XDR-TB costs. This is an attractive strategy because DR-TB treatment facilities in South Africa are severely overburdened [3] and there are often long waiting times for limited bed space thus facilitating community transmission. Indeed, in some areas like the Northern Cape out-patient treatment of XDR-TB has already begun. Furthermore, lack of proper infection control facilities in provinces like Kwa-Zulu Natal results in nosocomial transmission, which may be responsible for almost half of all XDR-TB cases in that province [42]. Given that approximately 50% of patients meet these criteria XDR-TB-related costs can be reduced by ∼$6930 per case. Based on notified XDR-TB cases in 2010, this represents a total savings of US$5.1 million per annum (∼2% of the National TB budget and ∼7% of the estimated DR-TB budget). In addition to the potential direct cost savings, a decentralized strategy would free up bed space for patients with more extensive disease and encourage treatment adherence among patients with limited access to these centralized facilities. Decentralized MDR-TB management, currently performed in some provinces in South Africa, is a cost effective option in other resource-poor settings as shown in our analysis and elsewhere [17]. However, implementation of such a strategy remains contentious as these patients need to be closely monitored, (particularly for capreomycin-related electrolyte abnormalities) and educated in proper infection control practices. Treatment default is also an issue which, in MDR-TB outpatients, can be up to 20% [20].

There are a number of limitations to our analysis. Our study is a simple cost analysis rather than a cost-effectiveness study, which also evaluates effectiveness of a management strategy. However, the purpose of our study was to report the costs of DR-TB in South Africa rather than compare the cost-effectiveness of different management strategies, which should be the focus of future studies. The use of cost data from Brooklyn Chest Hospital BCH is representative of the Western Cape but may not be reflect the rest of South Africa as hospitalization and treatment facility costs, as well as treatment regimens and some treatment policies for DR-TB, can vary slightly across provinces. However, we used standardized diagnosis and treatment protocols in our analysis, according to South African national TB guidelines, and varied the parameters that tend to be different across the provinces in the sensitivity analysis. We used treatment outcomes from published retrospective cohort studies due to lack of accurate surveillance data and to generalize our results for South Africa. However, we realize these results may not accurately reflect current disease outcomes. While decentralized MDR-TB treatment is now the current policy in South Africa, it is not fully implemented across all provinces and our assumption of predominant outpatient care for these patients may underestimate the cost of MDR-TB. However, we conducted a sensitivity analysis using a higher estimate of MDR-TB patients hospitalised (70%) and reported the per patient and total national costs using this estimate. Indeed, an increased incidence of inpatient care among MDR-TB patients does increase the DR-TB costs. However, our analysis calculates DR-TB costs based on adherence to current national DR-TB management guidelines (which recommends outpatient care for most MDR-TB cases) rather than the specific treatment practices in other provinces. Strict implementation of decentralized MDR-TB care is likely to become widespread across South Africa in the near future. ADRs are difficult to cost as the frequency and types of ADR varies widely among different cohort studies and one patient may experience more than one ADR several times during the course of treatment. Additionally, HIV infected individuals are likely to have a higher frequency of ADRs due to additional drug interaction with ARVs. As such, ADR costs may be underestimated. Total national TB costs refers to costs associated with diagnosis and treatment of confirmed TB cases and excludes certain costs, such as those associated with diagnosis of non TB cases. As such these costs may also be underestimated. Our decentralized model for XDR-TB did not include detailed costs associated with increased transmission or infection control. As such, the cost-saving of this model is likely to be overestimated. Future setting-specific detailed cost-feasibility studies are now needed to determine the practicality of implementing this model. Societal costs such as patient-related costs were not included as it is difficult to accurately capture patients’ out of pocket expenses and their socio-economic status during the time of illness. It is likely that such costs will be substantial given the long hospitalization periods, and extensive morbidity and mortality associated with DR-TB. Assessment of these costs should be the focus of future cost-analysis studies.

In conclusion, DR-TB in South Africa is extremely expensive with a small number of DR-TB cases disproportionately consuming a large chunk of the total NTP budget. Based on South African National DR-TB guidelines, the current cost of XDR-TB management is 103 times greater than DS-TB, the majority of which is attributable to the cost of hospitalization. Implementation of decentralized XDR-TB care is a viable option and can reduce these costs but will require intensive follow up and appropriate infection control measures to be put into place to minimise disease transmission.

## Supporting Information

Table S1 **Frequency and duration of hospitalization, outpatient/clinic visits, treatment and diagnostic/monitoring tests during the period of treatment for drug sensitive tuberculosis, multi-drug resistant tuberculosis and extensively drug-resistant tuberculosis according to the South African Drug Resistant TB guidelines.** The reported frequencies refer to the period from diagnosis till the end of treatment.
(DOCX)

## References

[1] World Health Organization (2011) WHO Report 2011: Global Tuberculosis Control. (2011) Available: http://www.who.int/tb/publications/global_report/en/. Accessed 2012 Oct 20.

[2] United Nations (2011) The UN Millenium Development Goals Report 2011 (2011) Available: www.un.org/millenniumgoals/reports.shtml.Accessed 2012 Mar.

[3] Dheda K, Migliori GB (2012) The global rise of extensively drug-resistant tuberculosis: is the time to bring back sanatoria now overdue? Lancet 379: 773–775. 22033020 10.1016/S0140-6736(11)61062-3

[4] Dheda K, Warren RM, Zumla A, Grobusch MP (2010) Extensively drug-resistant tuberculosis: epidemiology and management challenges. Infect Dis Clin North Am 24: 705–725. 20674800 10.1016/j.idc.2010.05.001

[5] South African Department of Health (2011) Management of Drug-Resistant Tuberculosis: Policy Guidelines (2011) Available: www.kznhealth.gov.za/pharmacy/ptc/MDR2011.pdf.Accessed 2012 Oct 3.

[6] Dheda K, Shean K, Zumla A, Badri M, Streicher EM, et al (2010) Early treatment outcomes and HIV status of patients with extensively drug-resistant tuberculosis in South Africa: a retrospective cohort study. The Lancet 375: 1798–1807. 10.1016/S0140-6736(10)60492-8 20488525

[7] Kvasnovsky CL, Cegielski JP, Erasmus R, Siwisa NO, Thomas K, et al (2011) Extensively Drug-Resistant TB in Eastern Cape, South Africa: High Mortality in HIV-Negative and HIV-Positive Patients. J Acquir Immune Defic Syndr 57: 146–152. 21297482 10.1097/QAI.0b013e31821190a3

[8] O'Donnell MR, Padayatchi N, Master I, Osburn G, Horsburgh CR (2009) Improved early results for patients with extensively drug resistant tuberculosis and HIV in South Africa. Int J Tuberc Lung Dis 13: 855–861. 19555535 PMC2855970

[9] Jacobson KR, Tierney DB, Jeon CY, Mitnick CD, Murray MB (2010) Treatment outcomes among patients with extensively drug-resistant tuberculosis: systematic review and meta-analysis. Clin Infect Dis 51: 6–14. 20504231 10.1086/653115 PMC4013786

[10] World Health Organization (2011) Tuberculosis Country Profiles: South Africa (2011) Available: http://www.who.int/tb/country/data/profiles/en/index.html.Accessed 2012 Jul.

[11] Meyer-Rath G, Schnippel K, Long L, MacLeod W, Sanne I, et al (2012) The impact and cost of scaling up GeneXpert MTB/RIF in South Africa. PLoS One 7: e36966. 22693561 10.1371/journal.pone.0036966 PMC3365041

[12] Oxlade O, Falzon D, Menzies D (2012) The Impact and Cost-effectiveness of Strategies to Detect Drug Resistant Tuberculosis. Eur Respir J 39: 626–634. 21828030 10.1183/09031936.00065311

[13] Resch SC, Salomon JA, Murray M, Weinstein MC (2006) Cost-effectiveness of treating multidrug-resistant tuberculosis. PLoS Med 3: e241. 16796403 10.1371/journal.pmed.0030241 PMC1483913

[14] Suarez PG, Floyd K, Portocarrero J, Alarcon E, Rapiti E, et al (2002) Feasibility and cost-effectiveness of standardised second-line drug treatment for chronic tuberculosis patients: a national cohort study in Peru. Lancet 359: 1980–1989. 12076553 10.1016/S0140-6736(02)08830-X

[15] Tupasi TE, Gupta R, Quelapio MID, Orillaza RB, Mira NR, et al (2006) Feasibility and cost-effectiveness of treating multidrug-resistant tuberculosis: a cohort study in the Philippines. PLoS Med 3: e352. 16968123 10.1371/journal.pmed.0030352 PMC1564168

[16] White VLC, Moore-Gillon J (2000) Resource implications of patients with multidrug resistant tuberculosis. Thorax 55: 962. 11050268 10.1136/thorax.55.11.962 PMC1745633

[17] Fitzpatrick C, Floyd K (2012) A Systematic Review of the Cost and Cost Effectiveness of Treatment for Multidrug-Resistant Tuberculosis. Pharmacoeconomics 30: 63–80. 22070215 10.2165/11595340-000000000-00000

[18] Statistics South Africa (2012) Statistical Release P0141 - Consumer Price Index August 2012 (2012) Available: http://www.statssa.gov.za/publications/P0141/P0141August2012.pdf.Accessed 2012 Oct 22.

[19] United Nations (2011) Operational Rates of Exchange. (2011) Available: http://treasury.un.org. Accessed 2011 Dec.

[20] Brust JCM, Lygizos M, Chaiyachati K, Scott M, van der Merwe TL, et al (2011) Culture Conversion Among HIV Co-Infected Multidrug-Resistant Tuberculosis Patients in Tugela Ferry, South Africa. PloS one 6: e15841. 21253585 10.1371/journal.pone.0015841 PMC3017058

[21] Calver AD, Murray M, Strauss OJ, Streicher EM, Hanekom M, et al (2010) Emergence of increased resistance and extensively drug-resistant tuberculosis despite treatment adherence, South Africa. Emerg Infect Dis 16: 264–271. 20113557 10.3201/eid1602.090968 PMC2958014

[22] Farley JE, Ram M, Pan W, Waldman S, Cassell GH, et al (2011) Outcomes of Multi-Drug Resistant Tuberculosis (MDR-TB) among a Cohort of South African Patients with High HIV Prevalence. PloS one 6: e20436. 21799728 10.1371/journal.pone.0020436 PMC3142109

[23] Heller T, Lessells RJ, Wallrauch CG, Barnighausen T, Cooke GS, et al (2010) Community-based treatment for multidrug-resistant tuberculosis in rural KwaZulu-Natal, South Africa. Int J Tuberc Lung Dis 14: 420–426. 20202299

[24] Iddriss A, Reddy D, Padayatchi N, Reddi A (2011) Pulmonary resection for extensively drug-resistant tuberculosis in Durban, South Africa. Joint Meeting of 19th ASCVTS and 21st ATCSA. Phuket, Thailand. 10.1016/j.athoracsur.2012.03.072 PMC3567439 22633500

[25] Mehta U, Durrheim DN, Blockman M, Kredo T, Gounden R, et al (2008) Adverse drug reactions in adult medical inpatients in a South African hospital serving a community with a high HIV/AIDS prevalence: prospective observational study. Br J Clin Pharmacol 65: 396–406. 18070223 10.1111/j.1365-2125.2007.03034.x PMC2291259

[26] Nathanson E, Gupta R, Huamani P, Leimane V, Pasechnikov AD, et al (2004) Adverse events in the treatment of multidrug-resistant tuberculosis: results from the DOTS-Plus initiative. Int J Tuberc Lung Dis 8: 1382–1384. 15581210

[27] Schaaf HS, Moll AP, Dheda K (2009) Multidrug-and extensively drug-resistant tuberculosis in Africa and South America: epidemiology, diagnosis and management in adults and children. Clin Chest Med 30: 667–683, vii–viii. 10.1016/j.ccm.2009.08.019 19925960

[28] Seung KJ, Omatayo DB, Keshavjee S, Furin JJ, Farmer PE, et al (2009) Early outcomes of MDR-TB treatment in a high HIV-prevalence setting in Southern Africa. PloS one 4: e7186. 19779624 10.1371/journal.pone.0007186 PMC2746313

[29] Shean KP, Willcox PA, Siwendu SN, Laserson KF, Gross L, et al (2008) Treatment outcome and follow-up of multidrug-resistant tuberculosis patients, West Coast/Winelands, South Africa, 19922002. Int J Tuberc Lung Dis 12: 1182–1189. 18812049

[30] Sotgiu G, Ferrara G, Matteelli A, Richardson MD, Centis R, et al (2009) Epidemiology and clinical management of XDR-TB: a systematic review by TBNET. Eur Respir J 33: 871–881. 19251779 10.1183/09031936.00168008

[31] World Health Organization (2002) Guidelines for cost and cost-effectiveness analysis of tuberculosis control. (2002) Available: http://whqlibdoc.who.int/hq/2002/WHO_CDS_TB_2002.305b.pdf. Accessed 2012 Jun 18.

[32] Cleary SM, McIntyre D, Boulle AM (2006) The cost-effectiveness of antiretroviral treatment in Khayelitsha, South Africa–a primary data analysis. Cost Eff Resour Alloc 4: 20. 17147833 10.1186/1478-7547-4-20 PMC1770938

[33] World Health Organization (2011) Rapid Implementation of the Xpert MTB/RIF diagnostic test (2011) Available: http://whqlibdoc.who.int/publications/2011/9789241501569_eng.pdf.Accessed 2012 Oct 22.

[34] South African Department of Health (2009) National Tuberculosis Management Guidelines (2009) Available: http://familymedicine.ukzn.ac.za/Libraries/Guidelines_Protocols/TB_Guidelines_2009.sflb.ashx.Accessed 2012 Aug 12.

[35] World Health Organization (2006) Guidelines for the programmatic management of drug-resistant tuberculosis. (2006) Available: http://www.who.int/tb/challenges/mdr/programmatic_guidelines_for_mdrtb/en/index.html. Accessed 2012 Oct 21.

[36] Suh DC, Woodall BS, Shin SK, Hermes-De Santis ER (2000) Clinical and economic impact of adverse drug reactions in hospitalized patients. Ann Pharmacother 34: 1373–1379. 11144691 10.1345/aph.10094

[37] Arbex MA, Varella Mde C, Siqueira HR, Mello FA (2010) Antituberculosis drugs: drug interactions, adverse effects, and use in special situations. Part 2: second line drugs. J Bras Pneumol 36: 641–656. 21085831 10.1590/s1806-37132010000500017

[38] Schwartzman K, Menzies D (2000) Tuberculosis screening of immigrants to low-prevalence countries. A cost-effectiveness analysis. Am J Respir Crit Care Med 161: 780–789. 10712322 10.1164/ajrccm.161.3.9902005

[39] Pooran A, Booth H, Miller RF, Scott G, Badri M, et al (2010) Different screening strategies (single or dual) for the diagnosis of suspected latent tuberculosis: a cost effectiveness analysis. BMC Pulm Med 10: 7. 20170555 10.1186/1471-2466-10-7 PMC2837635

[40] Global Issues Health: High Drug Prices Hamper Drug-Resistant TB Treatment (2011) Available: http://www.globalissues.org/news/2011/06/17/10134.Accessed 2011 Apr.

[41] Theron G, Peter J, van Zyl-Smit R, Mishra H, Streicher E, et al (2011) Evaluation of the Xpert (R) MTB/RIF assay for the diagnosis of pulmonary tuberculosis in a high HIV prevalence setting. Am J Respir Crit Care Med 184: 132–140. 21493734 10.1164/rccm.201101-0056OC

[42] Basu S, Andrews JR, Poolman EM, Gandhi NR, Shah NS, et al (2007) Prevention of nosocomial transmission of extensively drug-resistant tuberculosis in rural South African district hospitals: an epidemiological modelling study. The Lancet 370: 1500–1507. 10.1016/S0140-6736(07)61636-5 PMC3711808 17964351

[43] World Health Organization (2005) Anti-tuberculosis drug resistance in the world: the WHO/IUATLD Global Project on Anti- Tuberculosis Drug Resistance Surveillance. Report no. 3. Prev-alence and trends. WHO/HTM/TB/2004.343. Geneva, Switzer-land (2005).

[44] South African Department of Health. Tuberculosis Strategic Plan for South Africa, 2007–2011. Available: http://www.info.gov.za/view/DownloadFileAction?id=72544. Accessed 2012 Apr 10.

