# Supplementary material for: What is the Cost of Diagnosis and Management of Drug Resistant Tuberculosis in South Africa?
Source: PLoS One. 2013 Jan 18;8(1):e54587. doi: 10.1371/journal.pone.0054587 (PMC3548831; doi:10.1371/journal.pone.0054587)
Supplement: Table S1 — Frequency and duration of hospitalization, outpatient/clinic visits, treatment and diagnostic/monitoring tests during the period of treatment for drug sensitive tuberculosis, multi-drug resistant tuberculosis and extensively drug-resistant tuberculosis according to the South African Drug Resistant TB guidelines. The reported frequencies refer to the period from diagnosis till the end of treatment. (DOCX) [file pone.0054587.s001.docx]

**Supporting Information:**

**What is the cost of diagnosis and management of drug resistant tuberculosis in South Africa?**

**Table S1:** Frequency and duration of hospitalization, outpatient/clinic visits, treatment and diagnostic/monitoring tests during the period of treatment for drug sensitive tuberculosis, multi-

drug resistant tuberculosis and extensively drug-resistant tuberculosis according to the South African Drug Resistant TB guidelines. The reported frequencies refer to the period from diagnosis till the end of treatment.

| **Component** | | **DS-TB** | | | | **MDR-TB** | | **XDR-TB** | |
| --- | --- | --- | --- | --- | --- | --- | --- | --- | --- |
|  |  | **Smear**  **positive converters** | **Smear**  **positive**  **non converters** | **Smear**  **negative** | **Retreatment** | **Culture converters** | **Culture**  **non converters** | **Culture converters** | **Culture**  **non converters** |
| Hospital, outpatient and clinic visit frequency | Hospital inpatient months (hospitalized patients only) | ND | ND | ND | ND | 4 months | 12 months | 6 months | 12 months |
|  | Hospital  outpatient visits | ND | ND | ND | ND | Once per month during  intensive phase, every 2 months in continuation  phase | | Twice per month during  intensive phase, every  month in continuation  phase | |
|  | Clinic visits  (check-up) | 1 at diagnosis,  2 during Rx | 1 at diagnosis,  3 during Rx | 1 at diagnosis, 3 during Rx | 1 at diagnosis, 4 during Rx | Once a month for  monitoring ADRs (outpatients only) | | Once a month for  monitoring ADRs  (outpatients only) | |
|  | DOTS clinic visits  (administering drugs) | 1st 2 weeks  of Rx | 1st 2 weeks  of Rx | 1st 2 weeks  of Rx | daily during intensive phase | Daily for duration of Rx  (outpatients only) | | Daily for duration of Rx (outpatients only) | |
| Treatment regimen and duration | Length of  intensive phase | 2 months | 3 months | 2 months | 3 months | 6 months | 12 months | 6 months | 12 months |
|  | Intensive phase  regimen | R-H-Z-E | R-H-Z-E | R-H-Z-E | R-H-Z-E-S | Km-Z-Mxf-Eto-Trd | | (Primary) Cm-Z-Mxf-Eto-Trd-PAS-Cfz  (Acquired) Cm-Z-Mxf-Clm-Aug-  hdH -PAS-Cfz | |
|  | Length of continuation phase | 4 months | 4 months | 4 months | 5 months | 18 months | - | 18 months | 3 months |
|  | Continuation phase  regimen | R-H | R-H | R-H | R-H-E | Z-Mxf-Eto-Trd | | (Primary) Z-Mxf-Eto-Trd-PAS-Cfz  (Acquired) Z-Mxf-Clm-Aug-  hdH -PAS-Cfz | |
| Diagnostic and monitoring test frequency | Sputum smear microscopy | 2 at diagnosis, 4 during Rx | 2 at diagnosis, 5 during Rx | 2 at diagnosis, 5 during Rx | 2 at diagnosis, 5 during Rx | At diagnosis and  monthly | | At diagnosis and  monthly | |
|  | Sputum  liquid culture | ND | After 2 months  of Rx | At diagnosis | At diagnosis | At diagnosis and  monthly | | At diagnosis and  monthly | |
|  | 1st line DST | ND | ND | ND | At diagnosis | At diagnosis | | At diagnosis | |
|  | 2nd line DST | ND | ND | ND | ND | At diagnosis | At diagnosis and after 6 months of Rx | At diagnosis | At diagnosis and after  6 months of Rx |
|  | Chest X-ray | ND | ND | at diagnosis | at diagnosis | At diagnosis and  every 6 months | | At diagnosis and  every 6 months | |
|  | Full blood count | ND | ND | ND | ND | Baseline (every 6 months  in HIV-infected) | | Baseline (every 6 months  in HIV-infected) | |
|  | Urea | ND | ND | ND | ND | Baseline | | Baseline | |
|  | Kidney (creatinine, potassium) | ND | ND | ND | ND | Monthly during  intensive phase | | 1.5 per month during  intensive phase | |
|  | Liver (ALT, AST, Bilirubin) | ND | ND | ND | ND | Every 3 months | | Every 3 months | |
|  | TSH | ND | ND | ND | ND | Every 6 months | | Every 4 months | |
|  | Audiogram | ND | ND | ND | ND | Monthly during injectable  phase and every 3 months  in continuation phase | | Monthly during injectable  phase and every 3 months  in continuation phase | |
|  | CD4 count (HIV only) | Once during Rx period | | | | Every 6 months | | Every 6 months | |
|  | Viral load (HIV only) | Once during Rx period | | | | Every 6 months | | Every 6 months | |

ND – not routinely done, R - Rifampicin, H - Isoniazid, Z - Pyrazinamide, E - Ethambutol, S - Streptomycin, Km – Kanamycin, Mxf - Moxifloxacin, Eto - Ethionamide, Trd – Terizidone, Cm - Capreomycin, PAS – para-amionsalicylic acid, Cfz – Clofazimine, Clm - Clarithromycin, Aug - Augmentin, hdH - high dose Isoniazid

DST – Drug susceptibility test, AST - aspartate aminotransferase, ALT - alanine aminotransferase, TSH – Thyroid stimulating hormone, Rx – Treatment, DOTS – Directly Observed Treatment Short Course, ADR - Adverse drug reaction
